# Analysis of the function of *E. coli *23S rRNA helix-loop 69 by mutagenesis

**DOI:** 10.1186/1471-2199-6-18

**Published:** 2005-07-29

**Authors:** Aivar Liiv, Diana Karitkina, Ülo Maiväli, Jaanus Remme

**Affiliations:** 1 Estonian Biocentre, Riia 23, 51010 Tartu, Estonia; 2 Institute of Molecular Biology and Cell Biology, Tartu University, Riia 23, 51010 Tartu, Estonia; 3 Clinic for Neurology, Leipziger Str. 44, D-39120 Magdeburg, Germany

## Abstract

**Background:**

The ribosome is a two-subunit enzyme known to exhibit structural dynamism during protein synthesis. The intersubunit bridges have been proposed to play important roles in decoding, translocation, and the peptidyl transferase reaction; yet the physical nature of their contributions is ill understood. An intriguing intersubunit bridge, B2a, which contains 23S rRNA helix 69 as a major component, has been implicated by proximity in a number of catalytically important regions. In addition to contacting the small ribosomal subunit, helix 69 contacts both the A and P site tRNAs and several translation factors.

**Results:**

We scanned the loop of helix 69 by mutagenesis and analyzed the mutant ribosomes using a plasmid-borne IPTG-inducible expression system. We assayed the effects of 23S rRNA mutations on cell growth, contribution of mutant ribosomes to cellular polysome pools and the ability of mutant ribosomes to function in cell-free translation. Mutations A1912G, and A1919G have very strong growth phenotypes, are inactive during *in vitro *protein synthesis, and under-represented in the polysomes. Mutation Ψ1917C has a very strong growth phenotype and leads to a general depletion of the cellular polysome pool. Mutation A1916G, having a modest growth phenotype, is apparently defective in the assembly of the 70S ribosome.

**Conclusion:**

Mutations A1912G, A1919G, and Ψ1917C of 23S rRNA strongly inhibit translation. Mutation A1916G causes a defect in the 50S subunit or 70S formation. Mutations Ψ1911C, A1913G, C1914A, Ψ1915C, and A1918G lack clear phenotypes.

## Background

**Figure 1:**
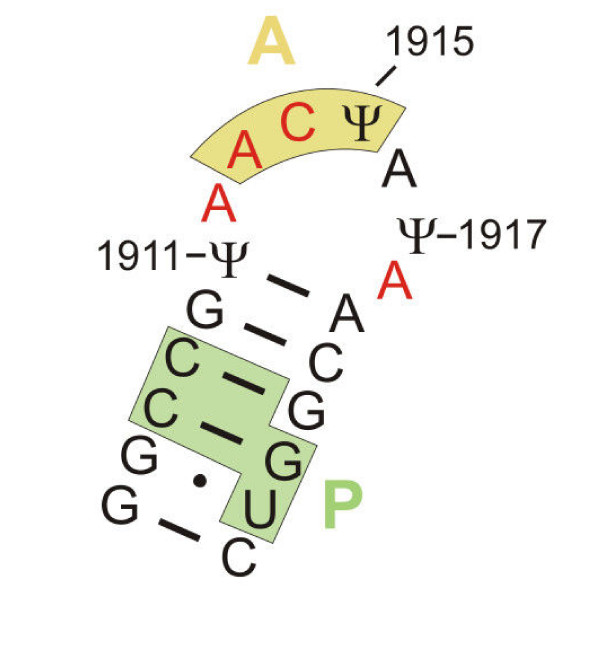
**A scheme of E. coli 23S rRNA helix 69. **Nucleotides implicated in contacts with 30S subunit are in red [1,3,6]. Proposed contact areas with A and P site tRNAs [1] are shown in yellow and green boxes. Numbers of the pseudouridine residues are indicated according to standard *E. coli *23S rRNA numeration.

High-to-medium-resolution structures of the ribosome have by their ability to generate structure-based functional hypotheses radically changed the way the ribosome is studied. One of the more intriguing results that has come from structural studies is the extraordinary number of roles attributed to a single 19 nt helix-loop, H69 of 23S rRNA (Fig 1). Crystallographic studies of the *Thermus thermophilus *ribosome [1] and cryo-EM studies of *E. coli *ribosomes [2,3] have made it evident that H69 is a component of both the A and P sites with an ability to simultaneously contact two tRNAs. It contacts the D-stem and D-stem junction of the A site tRNA by the loop residues 1913–1915 and the same parts of the P site tRNA by backbone-backbone interactions with stem nucleotides 1908, 1909, 1922 and 1923 [1] (Fig 1). In addition, H69 loop residues 1912, 1913, 1914 and 1918 contact 16S rRNA H44, thus forming the intersubunit bridge B2a [1,2]. Chemical cross-linking and footprinting data further corroborates the close proximity of H69 to intersubunit contact area [4,5]. The importance of H69 in subunit association is emphasized by the recent finding that DMS-modifications of A1912 or A1918 (but not of A1913) abolish 70S formation in an *in vitro *test system [6]. Also, hydroxyl-radical footprinting of the anti-subunit-association factor IF3 on the 30S subunit implicates IF3 binding to the region that is occupied by the loop of H69 in 70S ribosomes [7], suggesting that disallowing of the bridge B2a may be important for keeping the subunits separate before correct initiation of translation.

Since H69 adopts a different conformation in 50S subunits and 70S ribosomes, it has to change conformation upon 30S binding [1,8]. Conformational flexibility of H69 may also be important in translocation since it is hard to imagine tRNA movement from A to P site with H69 stuck in its path. An active role for H69 in translocation has been proposed [9] but has not yet been experimentally tested. In addition to interactions with tRNAs and the 30S subunit, contacts of H69 with various A site substrates have been proposed. Based on cryo-EM reconstitution of the ribosome with bound aa-tRNA-EF-Tu-GDP-kirromycin, Valle et al. speculate that tRNA contacts with H69 might actively promote the observed kink in tRNA structure [10]. Cryo-EM studies have also led to proposals of H69 contacts with eEF2 [11], RF2 [12], RF3 [13], RRF [14] and SmpB in the Ala-tmRNA-SmpB-EF-Tu-kirromycin complex [15].

Another interesting feature of the H69 is its three pseudouridines at positions 1911, 1915 and 1917 [16]. They are synthesized by a single synthase, RluD, which is the only pseudouridine synthase in *E. coli *whose deletion leads to a strong growth defect [17]. Defective RluD function leads to impaired ribosome assembly [18]. This observation suggests that H69 actively promotes the process of ribosomal large subunit assembly.

O'Connor and Dahlberg selected three mutations in H69 (ΔA1916, insertion of two adenosines after A1916, and C1914U) that cause increased +1 and -1 frameshifting and read-through of all three stop codons [19]. Here we mutate each residue in the loop of H69 and analyze the growth phenotypes, assembly of the mutant ribosomes, their incorporation into polysomes and activities in poly-uridine-directed poly-phenylalanine synthesis. The results obtained in this work point to residues A1912, A1916, Ψ 1917 and A1919 as important for correct functioning of the *E. coli *ribosome.

## Results

### Experimental design

Because of the crucial nature of protein synthesis for cellular viability and the perceived importance of 23S rRNA helix 69 for correct functioning of the ribosome, its mutations are likely to be lethal. Therefore, we used inducible expression to study *in vivo *phenotypes of mutations in 23S rRNA. Mutated 23S rRNA genes were expressed from the plasmid ptBsB under the control of IPTG-inducible tac promoter [20]. In order to be able to quantify the fraction of mutant 23S rRNA in ribosomes and to functionally differentiate between plasmid-borne and chromosomally encoded ribosomes during *in vitro *translation, the single mutations were combined with the second site mutation A1067U in the plasmid ptBsB1067T. This mutation confers resistance to thiostrepton during cell free translation [21]. Therefore, A1067U enables to discriminate the activity of chromosomally encoded wild-type ribosomes from mutant ribosomes containing plasmid-encoded 23S rRNA. 30–40% of the cellular ribosome pool contain mutant 23S rRNA [22]. We constructed the following mutations in the loop of H69: Ψ1911C, A1912G, A1913G, C1914A, Ψ1915C, A1916G, Ψ1917C, A1918G and A1919G.

### Effect of mutations on cell growth

**Figure 2:**
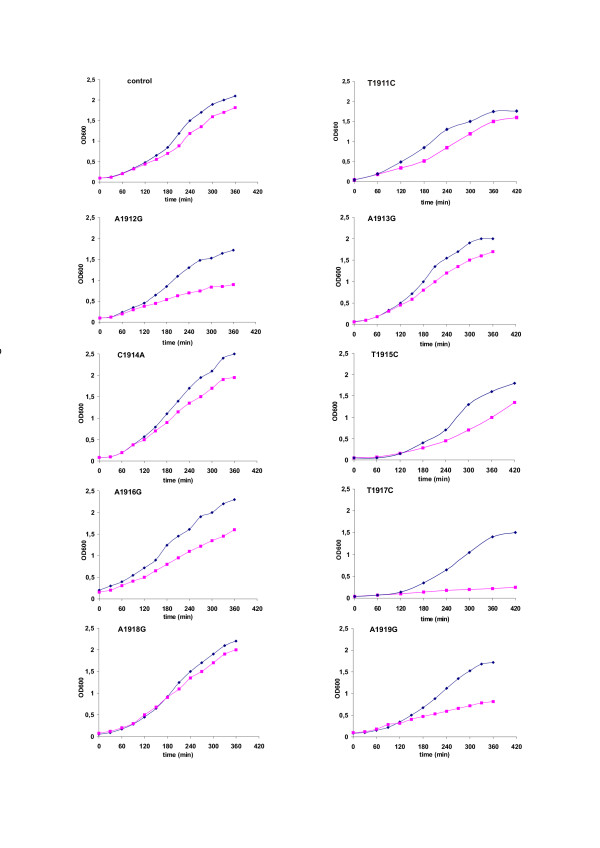
**Growth of XL-1 cells expressing mutant 23S rRNA. **The expression of the mutant rRNA was induced at O.D._600 _= 0.05 (0 min time point) by addition of IPTG. Y-axis shows the optical densities of the cultures at time points. Control plasmid is ptBsB1067T. Density of the bacterial culture grown in the absence of IPTG is shown by blue circles and in the presence of IPTG is shown by red squares.

Mutant 23S rRNA expression was induced with 1 mM IPTG in XL-1 cells growing in rich liquid media at 37°C in the early exponential growth phase. The plasmid, carrying 23S rRNA gene with the single mutation A1067U, was used as a control. The expression of 23S rRNA genes containing mutations A1912G, Ψ1917C and A1919G resulted in very strong growth inhibition 2–3 hrs post-induction leading to a complete cessation of cell growth well below the cell densities, which were reached by uninduced cultures (Fig 2). Induction of 23S rRNA variants carrying mutations Ψ1915C and A1916G resulted in modestly increased doubling times but nevertheless allowed the cultures to reach maximal cell densities similar to uninduced cultures (Fig 2). Mutations Ψ1911C, A1913G, C1914A and A1918G had little or no effect on growth (Fig 2).

### Incorporation of mutant 23S rRNA into poly-ribosomes

Quantitative monitoring of the proportion of mutant 23S rRNAs in translating polysomes, initiating 70S ribosomes and translationally idle 50S subunits enables us to assign defects in translation to the initiation phase (when mutant rRNA is under-represented in 70S and over-represented in 50S fractions) or the elongation phase (when mutant rRNA is under-represented in polysomes) of translation. Furthermore, possible changes in relative sizes of the polysome, 70S or 50S fractions can provide useful information on translational competency of the mutant cells. In addition, changes in the overall shape and number of the peaks can point to defects in ribosome assembly.

The marker-mutation A1067U was used to determine the fraction of mutant 23S rRNA. Ribosomes were fractionated by 15%-40% sucrose gradient centrifugation and rRNA was extracted from tetrasome, trisome, disome, 70S and 50S fractions. Relative proportions of plasmid-encoded and chromosomally encoded rRNAs were determined by a standard primer extension assay [23].

**Figure 3:**
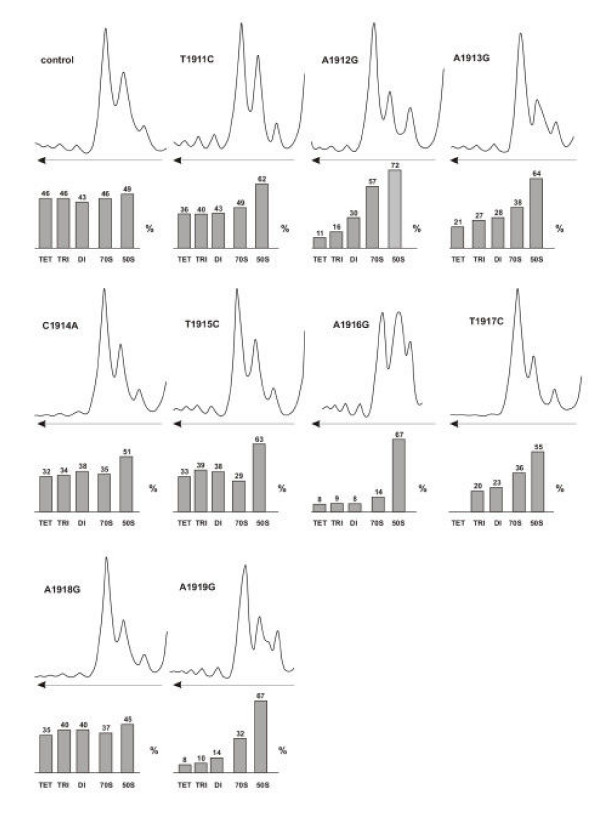
**Distribution of plasmid encoded 23S rRNA in ribosomal fractions. **Mutant plasmids were expressed in the strain XL1-Blue and ribosomes were fractionated by sucrose gradient centrifugation. The percentage of plasmid-encoded 23S rRNA in the ribosomal fractions was determined by RNA sequencing. The gradient profile and the percentages of the plasmid-encoded 23S rRNA in the corresponding gradient fractions is shown for each mutant. The arrow shows the direction of sedimentation. TET- tetrasomes, TRI- trisomes, DI- disomes. Control plasmid is ptBsB1067T.

Expression of the single mutation A1067U 23S rRNA variant results in around 45% of the polysome and 70S pools consisting of mutant ribosomes (Fig 3). 23S rRNA variant A1912G is under-represented in polysomes, but not in the 70S ribosomes (Fig 3). Its progressively larger deprivation in larger polysomes (30% presence in disomes, 15% in trisomes, and 10% in tetrasomes) is consistent with defects in the elongation phase of translation of the mutant ribosomes rather than, for example, in a late initiation step.

Mutation A1913G exhibits a modest counter-selection of mutant ribosomes in the polysome fractions reaching approximately two-fold deprivation in the tetrasomes (Fig 3).

23S rRNA variant Ψ1917C exhibits a relatively modest fractional deprivation in polysomes and in 70S ribosomes (Fig 3). Interestingly, it leads to a large reduction in the amount of polysomes; indeed so large that we were unable to collect tetrasomes for analysis. Therefore, expression of this mutant must reduce the ability of the wild-type ribosomes to engage in translation.

Mutation A1919G leads to a modest deprivation of plasmid borne 23S rRNA in the 70S ribosomes, in addition to a large progressive deprivation in the polysomes fractions (Fig 3). This is, once again, suggestive of a mostly elongation-level defect in translation by the mutant ribosomes. In addition, expression of the 23S rRNA variant A1919G led to appearance of an extra gradient peak, corresponding to particles sedimenting approximately as 40S (Fig 3). However, the amount of the 40S particles varied widely between experiments from nonexistent to nearly the levels of the 50S subunits (data not shown). In spite of the variable results we tentatively suggest that the mutation A1919G affects 50S subunit assembly.

23S rRNA variant A1916G exhibits a large deprivation in 70S ribosomes and is nearly absent in polysomes (Fig 3). In addition, 23S rRNA variant A1916G reproducibly exhibited enlarged and widened 50S peak, suggestive of conformational heterogeneity in the 50S population (Fig 3). Conformational heterogeneity can be caused by a defect in ribosomal large subunit assembly. It is possible that the transition A1916G confers a defect of ribosome large subunit assembly. On the other hand, lack of 23S rRNA variant A1916G in the 70S and polysome fractions can be caused by a defect in association with the 30S subunit, which in turn can cause an initiation defect.

Expression of mutations Ψ1911C, C1914A, Ψ1915C or A1918G did not lead to significant changes in the fraction of mutant ribosomes or in the appearances of the gradient profiles (Fig 3).

### Cell-free translation of poly(U) by mutant ribosomes

**Figure 4:**
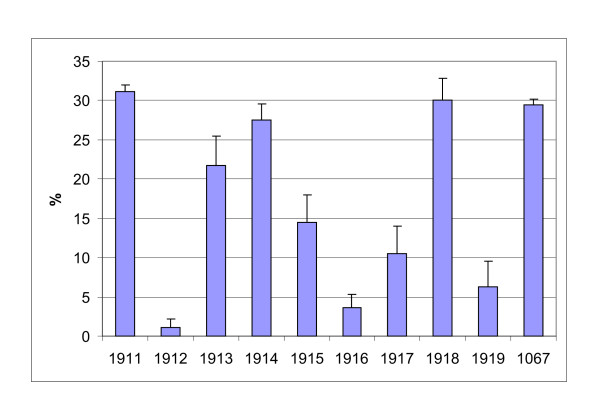
**Cell-free Poly(U)-dependent protein synthesis activity in the presence of thiostrepton. **Ratio of protein synthesis in the presence and absence of the drug is given as resistance. Standard deviations from 2–5 independent experiments are shown. Control plasmid is ptBsB1067T.

For *in vitro *translation, tight-couple 70S ribosomes were isolated from induced XL1 lysates by sucrose gradient ultracentrifugation. The second site mutation, A1067U, confers thiostrepton resistance to plasmid-borne 50S ribosomes enabling studies of the cell free translation of mutant ribosomes through inactivation of the wild-type ribosomes by thiostrepton. In the presence of five-fold molar excess of thiostrepton, poly(U)-directed translation of wild-type ribosomes was inhibited by 97–99% [22]. The ribosomes isolated from induced cells that harbor plasmid-encoded A1067U mutant 23S rRNA exhibited 30% thiostrepton resistance during poly(U) translation (Fig 4). If A1912G, A1916G or A1919G mutation was added as the second mutation to the A1067U, thiostrepton-resistance dropped to nearly zero (Fig 4). This means that ribosomes harboring mutations at positions 1912, 1916 and 1919 are completely inactive in the poly(U)-directed translation system. Mutation Ψ1917C causes a three-fold reduction in poly(Phe) synthesis of the mutant ribosomes (Fig 4). Mutation Ψ1915C cause a two-fold reduction in cell-free translation capability of the mutant ribosomes (Fig 4). Mutation A1913G exhibits slightly reduced levels in poly(U) translation. Mutations Ψ1911C, C1914A and A1918G have no effect on poly(U) translation.

## Discussion

We have dissected the function of the helix-loop 69 of 23S rRNA by subjecting mutants of its loop residues to various functional tests. The results of assays of cell growth, polysome incorporation, and cell-free translation pointed to the same mutations (A1912G, A1916G, Ψ1917C and A1919G) as seriously compromised. However, the phenotypes of the aforementioned mutations fall into three distinct types.

First, mutations at positions 1912 and 1919 are defective in the elongation phase of protein synthesis, both having reduced amounts of mutant ribosomes in the polysomes, but are abundant in the 70S ribosomes. The gradual decrease of the A1912G and A1919G ribosomes in successive polysomal fractions is consistent with placement of one mutant ribosome per mRNA. This can happen when the mutant ribosomes are able to initiate translation but cannot undergo into elongation phase. The mutant ribosomes are also completely inactive during *in vitro *translation, showing that the under-representation of mutant ribosomes in cellular polysomes is a direct consequence of the inactivity of the mutant ribosomes. Although mutant ribosomes are largely excluded from the polysomes, the total amount of polysomes in the relevant sucrose gradient fractions is not appreciably reduced (Fig 3). It should be noted that expression of 30–40% of mutant ribosomes in the background of wild-type ribosomes rapidly leads to a complete cessation of cell growth. It is likely that this pseudo-dominant phenotype is caused by "choking" of the translation by freezing the ribosomes (both mutant and wt) on the cellular mRNAs.

Second, as indicated by the widened 50S peak in the sucrose gradient, ribosomes carrying the mutation A1916G are structurally heterogeneous and thus apparently defective in the assembly of mutant 50S subunits. This conjecture is further supported by the strongly reduced activity of the mutant ribosomes in cell free translation and by their strong counter-selection in the 70S ribosome pool. Yet this mutation leads only to a modest retardation in cell growth. Therefore, complete inactivation of the mutant ribosomes in the wild-type background is not in itself detrimental to cell growth.

Third, the Ψ1917C mutation leads to relatively modest effects in cell free translation and in the fractions of mutant ribosomes in the polysomes. Therefore, unlike for A1912G and A1919G, the induction of Ψ1917C-carrying 23S rRNA does not freeze the ribosomes (mutant or wild-type) on mRNAs. Yet it leads to a complete stop in cell growth 3–4 hours after the induction of mutant 23S rRNA synthesis. The Ψ1917C is also the only mutation that leads to a clearly reduced polysome pool 2 hours after induction. We believe that this reduction in the ability of both mutant and wild-type ribosomes to enter the polysome pool could explain the observed drastic growth defect by strong reduction in the cellular protein synthesis levels. Such an effect could, in principle, be achieved by sequestering of an essential factor for translation by the mutant ribosomes.

A number of nucleotides in the loop of helix 69 have been implicated in binding of the A site tRNA (A1913-Ψ1915) and as components of the intersubunit bridge B2a (A1912-C1914, A1918) based on structural [1,3] and modification interference [6] studies (Fig 1). Surprisingly, of the aforementioned five nucleotides, only the mutation A1912G exhibited a strong phenotype. The DMS-modification of the N1 position of A1912 was previously shown to be detrimental to 70S ribosome formation *in vitro *[6]. However, the A1912G mutation seems to exert its strong effect on translation at the level of elongation, rather than by inhibiting 70S ribosome formation (Fig 3). Therefore, we failed to confirm the functional importance of any of the proposed H69 contacts with tRNA or the SSU by the mutagenesis approach. This is reminiscent of the results of O'Connor and Dahlberg who disrupted the stem of H69 by introducing a C1909:C1921 mismatch and found no growth effect or defects in translation [19]. Yet, this disruption should fall squarely in the middle of the H69 backbone-to-backbone contact area with the P site bound tRNA [1] (Fig 1). Also, of the three conserved pseudouridines in H69, only mutation of the Ψ1917 has a strong effect. Notably, out of three pseudouridine residues in this region, only the Ψ1917 is universally conserved [16]. Thus, Ψ1917 is likely to have a unique function.

Sucrose gradient pattern suggested that the mutation A1916G affects 50S assembly. As the H69 forms a spindle-like structure on the interface side of the large subunit [1,8], it is difficult to imagine how the mutation in the loop region could affect 50S structure, and thereby its assembly. On the other hand, A1916G transition can affect ribosomal subunit interaction. It is possible that association of the subunits is important for final maturation of the 50S subunits.

## Conclusion

We scanned the loop of helix 69 by mutagenesis and analyzed the mutant ribosomes using a plasmid-borne IPTG-inducible expression system. We assayed the effects of 23S rRNA mutations on cell growth, contribution of mutant ribosomes to cellular polysome pools and the ability of mutant ribosomes to engage in cell-free translation. Mutations A1912G, and A1919G have very strong growth phenotypes, are inactive during *in vitro *protein synthesis and are under-represented in the polysomes. Mutation Ψ1917C has a very strong growth phenotype and leads to a general depletion of the cellular polysome pool. Mutation A1916G, having a modest growth phenotype, is apparently defective in the assembly of the mutant 50S subunits or in the 70S formation.

## Methods

### Plasmids, strains and mutagenesis

The host strain for plasmids was *E. coli *XL1-Blue (supE44 hsdR17 recA1 endA1 gyrA46 thi relA1 lac^- ^F' [proAB^+ ^lacI^q ^lacZΔ M15 Tn10(tet^r^). Plasmid ptBsB1067T [20,24] containing the BstE II-BamH I fragment of the *rrnB *operon (tRNA^Glu2^-23 S rRNA and the 5 S rRNA genes) under the control of the inducible *tac *promoter was used to construct the mutations. A single point mutation at position A1067 to T confers the thiostrepton resistance of plasmid borne ribosomes [21].

Site-directed mutagenesis was performed by the PCR-based approach of Mikaelian [25]. All PCR fragments were fully sequenced after cloning into the 23S rRNA gene in ptBsB1067U (the SalI-SacII fragment was replaced).

### Measurement of cell growth

Cell growth was measured at OD_600 _using *E. coli *strain XL-1 Blue containing mutant 23S rRNA genes in the plasmid pBsB1067U and a low copy-number plasmid pREP4, which expresses additional lac repressor protein [26]. Cells were grown at 37°C in rich liquid media (2xYT) with ampicillin (100 μg/ml) and kanamycin (50 μg/ml) and mutant 23S rRNA expression was induced with 1 mM IPTG. Culture densities were monitored for 12 hrs after induction.

### Preparation of the ribosomes and analysis of mutant rRNA content in polysomes

*E. coli *strain XL1-Blue transformed with the ptBsB1067T derivative plasmids were grown at 37° C in 2xYT medium (16 g/l tryptone, 10 g/l yeast extract, 5 g/l NaCl) supplemented with ampicillin (100 μg/ml). Ribosomes were isolated from cells after induction with IPTG (1 mM) at A_600 _= 0.2 for 2 hours. Bacteria were collected by low-speed centrifugation and resuspended in lysis buffer (16% sucrose (w/v), 6 mM MgCl_2_, 60 mM NH_4_Cl, 60 mM KCl, 50 mM Tris-HCl pH-8.0, 6 mM β-mercaptoethanol). After addition of lysozyme (0.5 mg/ml final concentration) the cells were lysed by freezing and thawing 3 times. S-30 lysate was prepared by centrifugation at 12.000 g for 30 min in an SS34 rotor (Sorvall). The volume of the lysate was doubled with the buffer LLP (12 mM MgCl_2_, 60 mM NH_4_Cl, 60 mM KCl, 20 mM Tris-HCl pH-8.0, 6 mM β-mercaptoethanol), loaded onto a 5 ml sucrose cushion (20% sucrose, 12 mM MgCl_2_, 500 mM NH_4_Cl, 50 mM Tris-HCl pH-8.0, 6 mM β-mercaptoethanol) followed by the centrifugation for ω^2^t = 5.0 × 10^11 ^using a Beckman SW-41 rotor. Crude ribosomes were dissolved in buffer LLP and stored in small aliquots at -80°C.

For preparation of polysomes, 70 S ribosomes and 50 S ribosomes, the cell lysates were diluted 2 times with LLP buffer and loaded onto a 15–40% sucrose gradient in LLP buffer and centrifuged for ω^2^t = 3.5 × 10^11 ^in a Beckman SW-28 rotor. Polysomal, 70 S, 50 S, and 30 S gradient fractions were collected and precipitated with 2.5 volumes of ice-cold ethanol. rRNA was prepared using modified protocol of [27] For the extraction of rRNA ribosomes were dissolved in 200 μl water and 1 ml of PN solution (Qiagen, Cat. No. 19071) was added. Ribosomal proteins were extracted by vigorous shaking for 20 min at room temperature. 20 μl 50% silica suspension in water was added and RNA was bound for additional 10 min at room temperature with gentle mixing. Silica was pelleted by centrifugation at 6000 rpm for 30 sec and washed twice with 70% ethanol. RNA was eluted with 50 μl of water (10 min at room temperature). The proportion of plasmid-encoded 23S rRNA was determined by the modified primer extension protocol of Sigmund et al. [23] using the A1067T as the marker-mutation [22]. The resulting DNA fragments were resolved in 12% polyacrylamide-urea gel. Autoradiograms were digitalized using PhosphoImager (Molecular Dynamics) and quantified using the ImageQuant software (Molecular Dynamics).

### Poly(U)-directed protein synthesis

Poly(U) translation was performed essentially as described in Saarma and Remme [20]. Thiostrepton (Calbiochem) was dissolved in dimethylsulfoxside (DMSO) to 1 mM and used for inhibiting wild-type ribosomes. 0.5 A_260 _units of ribosomes were preincubated at 37°C for 15 min in the presence or absence of 7.5 μM thiostrepton and 0.02 mg poly(U) in 50 μl buffer LLP followed by the addition of 50 μl of factor mix containing 0.02 mg bulk tRNA (Boehringer Mannheim), 2 mM ATP, 0.5 mM GTP, 8 mM phosphoenolpyruvate (PEP), 2 μM pyruvate kinase, 0,01 mM [^14^C]Phe (150 cpm/pmol, Amersham) and 0.2 mg S-100 enzymes. After 30 min incubation at 37°C, reactions were stopped by addition of 1 ml 5% trichloroacetic acid (TCA) and heated for 20 min at 95° C. Precipitates were collected onto GF/A filters (Whatman) and counted for radioactivity. Thiostrepton resistance of the ribosomes was calculated by dividing TCA-insoluble radioactivity obtained in the presence of thiostrepton to that obtained in the absence of the drug.

## Authors' contributions

AL and DK did most of the experimental work and participated in planning and design of the experiments. ÜM participated in the analysis of mutant ribosomes and writing of the manuscript. JR conceived the study, participated in its design, and helped to write the manuscript.
